# 

**Published:** 1872-03

**Authors:** 


### TABLE OF CONTENTS.

ORIGINAL COMMUNICATIONS:

On Insufficient Vaccination. By Wm. Henry Cumming, M.D., Atlanta.. 671

Reflections on Uterine Disease, as Connected with Phthisis Pulmonalis. By Geo. M. McDowell, M.D., President of the Georgia Medical Association, Barnesville, Georgia.............................. 677

Atlanta Academy of Medicine. Jas. B. Baird, M.D., Reporter........ 683

Suspended Fœtal Animation—(Concluded.) By A. W. Griggs, M.D., West-Point, Georgia........................................ 701

Professional Olla-Podrida. By Robert Battey, M.D., Rome, Georgia 705
(Anomalous Pulse.—Chloral.—An Empty Stomach Can't Vomit.)

Cerebro-Spinal Meningitis. By J. G. Westmoreland, M.D., Professor of Materia Medica and Therapeutics in Atlanta Medical College..... 709

Effects of the White Sulphur Water of Greenbrier, Virginia, in Inebriation. By J. J. Moorman, M.D., Professor of Hygiene and Medical Jurisprudence in Washington University, Baltimore, etc........ 711

FOREIGN CORRESPONDENCE:

Supra-Pubic Puncture of the Bladder. By A. W. Calhoun, M.D..... 714

Syphilis-Corpuscles. (Selected).................................. 718

EDITORIAL AND MISCELLANEOUS:

Georgia Medical Association.......................................... 721

Georgia Lunatic Asylum.................................................. 723

Extract from the Minutes of the Georgia Medical Association...... . 724

Dr. A. W. Calhoun......................................................... 724

Quackery in Medical Journalism...................................... 724

Bibliographical.............................................................. 725

Middle Georgia Medical Society...................................... 730

Rush Medical College, Chicago........................................ 731

Annual Meeting of the Georgia Medical Association................ 732

Atlanta Medical College—Preparatory Course...................... 732

